# Mapping the implementation and challenges of clinical services for psychosis prevention in England

**DOI:** 10.3389/fpsyt.2022.945505

**Published:** 2023-01-03

**Authors:** Andrés Estradé, Tom John Spencer, Andrea De Micheli, Silvia Murguia-Asensio, Umberto Provenzani, Philip McGuire, Paolo Fusar-Poli

**Affiliations:** ^1^Early Psychosis: Interventions and Clinical-Detection (EPIC) Lab, Department of Psychosis Studies, Institute of Psychiatry, Psychology and Neuroscience, King's College London, London, United Kingdom; ^2^Department of Psychosis Studies, Institute of Psychiatry, Psychology and Neuroscience, King's College, London, United Kingdom; ^3^Outreach and Support in South London (OASIS) Service, South London and Maudsley NHS Foundation Trust, London, United Kingdom; ^4^Tower Hamlets Early Detection Service (THEDS), East London NHS Foundation Trust, London, United Kingdom; ^5^Department of Brain and Behavioral Sciences, University of Pavia, Pavia, Italy; ^6^National Institute for Health Research, Maudsley Biomedical Research Centre, South London and Maudsley NHS Foundation Trust, London, United Kingdom

**Keywords:** psychosis prevention, clinical high risk for psychosis, schizophrenia, psychosis, at-risk mental state (ARMS)

## Abstract

**Introduction:**

Indicated primary prevention of psychosis is recommended by NICE clinical guidelines, but implementation research on Clinical High Risk for Psychosis (CHR-P) services is limited.

**Methods:**

Electronic audit of CHR-P services in England, conducted between June and September 2021, addressing core implementation domains: service configuration, detection of at-risk individuals, prognostic assessment, clinical care, clinical research, and implementation challenges, complemented by comparative analyses across service model. Descriptive statistics, Fisher's exact test and Mann-Whitney *U*-tests were employed.

**Results:**

Twenty-four CHR-P clinical services (19 cities) were included. Most (83.3%) services were integrated within other mental health services; only 16.7% were standalone. Across 21 services, total yearly caseload of CHR-P individuals was 693 (average: 33; range: 4–115). Most services (56.5%) accepted individuals aged 14–35; the majority (95.7%) utilized the Comprehensive Assessment of At Risk Mental States (CAARMS). About 65% of services reported some provision of NICE-compliant interventions encompassing monitoring of mental state, cognitive-behavioral therapy (CBT), and family interventions. However, only 66.5 and 4.9% of CHR-P individuals actually received CBT and family interventions, respectively. Core implementation challenges included: recruitment of specialized professionals, lack of dedicated budget, and unmet training needs. Standalone services reported fewer implementation challenges, had larger caseloads (*p* = 0.047) and were more likely to engage with clinical research (*p* = 0.037) than integrated services.

**Discussion:**

While implementation of CHR-P services is observed in several parts of England, only standalone teams appear successful at detection of at-risk individuals. Compliance with NICE-prescribed interventions is limited across CHR-P services and unmet needs emerge for national training and investments.

## Introduction

Schizophrenia affects about 20 million people globally and is a top leading cause of health-related disability (1), with associated economic costs ranging from 0.02 to 1.65% of the gross domestic product (2) and severe disruption to the personal life of those affected (3). Schizophrenia-spectrum and other primary psychotic disorders have an estimated mean lifetime prevalence of 9.57 per 1,000 (4), and often begin during adolescence and young adulthood [meta-analytic peak age of onset at 20.5 years (5)]. Following a first-episode of psychosis (FEP), current antipsychotic treatments are limited at improving the causes, pathophysiology and course of schizophrenia (6). In consequence, primary indicated interventions (i.e., targeted at individuals with attenuated signs or symptoms of psychosis) have been incorporated into clinical practice to improve long-term outcomes from an earlier clinical stage (7–10). Preventive strategies became feasible following the introduction of the “at-risk mental state” (ARMS) or “clinical-high risk state” for psychosis (CHR-P) constructs (11, 12). Individuals meeting criteria for CHR-P are young [typically 14–35 years (10)], accumulate various known risk factors (13–15), and experience a deterioration in functioning (16) and neurocognition (17) as well as “attenuated psychotic symptoms” (APS) (18) or “brief limited intermittent psychotic symptoms” (BLIPS) (19–21). The CHR-P criteria is robustly associated with an enhanced risk of developing a psychotic disorder (OR: 9.32) (13) peaking at 2 years (cumulative risk: 0.25) following the initial assessment and continuing to slowly increase over time to a cumulative risk of 0.35 at 10 years (22, 23). Deficits in social cognition (17, 24), comorbid mental health issues such as anxiety and depression (25), and suicidal ideation (26) are prominent among CHR-P individuals. Over the long term, non-transitioning CHR-P individuals are affected by mental health problems or decreased functioning (27–29), including premature death (23), at higher rates than general population.

The CHR-P paradigm has extended across the world (30) through the implementation of specialized clinics that provide assessment, clinical monitoring, and interventions to CHR-P individuals (31), often within a non-stigmatizing community setting (32). Regional or national networks of CHR-P services have been established in England (33), Italy (34), Switzerland (35), and the United States (36, 37). CHR-P services can operate as “standalone” teams working independently from other generic mental health services, as “integrated” teams within broader services, for example within early intervention services for psychosis also offering care to FEP individuals, or under a “hub and spoke” model, which have been described elsewhere (38). Preventive care implemented by CHR-P clinic is contingent on detection of CHR-P individuals: this process is non-systematic, and determined by referrals upon suspicion of psychosis risk (31, 33). In this context, help-seeking behaviors among young people at-risk of psychosis is typically triggered not only by APS, but more likely by psychosocial dysfunction or affective symptoms (39).

Overall, the CHR-P represents one of the most established preventive paradigms in clinical psychiatry (10), having impacted national (40–43) and international (44) clinical guidelines. In the UK, the National Institute for Health and Care Excellence (NICE) (41, 42) recommended for young individuals at-risk for psychosis the provision of early assessment, regular monitoring of symptoms and functioning, alongside with individual cognitive-behavioral therapy (CBT), with or without family therapy, and support for comorbid mental health conditions, while antipsychotic medications are not recommended. However, earlier reports found inconsistencies among HCR-P services in the capacity to meet NICE-recommended interventions (45). The 2016 and 2020 Access and Waiting time Standards (46) ratified the importance of implementing CHR-P services nationwide but did not regulate how these services should be configured. Similar heterogeneity in service provision and delivery for CHR-P individuals is typically observed worldwide (30, 31). The aim of this study is to systematically analyses the implementation extent of CHR-P services across England, focusing on core implementation domains including service configuration, detection of at-risk individuals, prognostic assessment, clinical care, clinical research (10), and implementation challenges.

## Methods

### Survey procedure

An electronic audit of CHR-P services was developed, based on previous collaborative work conducted by the Pan-London Network for Psychosis-Prevention (PNP) (33), leveraging current clinical guidelines (41, 42) and global healthcare research (30, 31, 47). The audit was implemented using REDCap electronic data capture tools (48), hosted at King's College London. Email invitations were sent in May 2021 to CHR-P services and early intervention services taking care of individuals with a FEP, regional leads and other stakeholders. Dissemination of the audit was further supported by networking with NHS England and several early interventions networks and clinical academic sites in the England. Follow-up emails were sent in July 2021 and September 2021 to services not replying or providing incomplete responses. Overall, 107 early intervention services from 48 distinct NHS Trusts were targeted. Only services with a CHR-P clinical component were asked to complete the audit.

### Recorded variables

The audit included a combination of closed, multiple-choice, or open questions distributed across core implementation domains: (a) service configuration: (b) detection of at-risk individuals; (c) prognostic assessment; (d) clinical care; (e) clinical research (Supplementary Table 1). In addition, (f) implementation challenges across the above domains were evaluated *via* open-ended questions.

### Data analysis

Data was analyzed with descriptive statistics using IBM's Statistical Package for the Social Sciences (SPSS) v.27 software, including frequencies for categorical variables, and mean, standard deviation (SD) and median for continuous variables. Comparative analyses were conducted to stratify findings according to service configuration (i.e., integrated vs. standalone vs. hub and spoke), including key variables belonging to the detection of at-risk individuals (outreach and service promotion activities, online presence, availability of self-referral, caseload of CHR-P individuals), prognostic assessment (systematic collection of outcome measures, regularly collected outcomes), clinical care (duration of service provision, NICE-compliant intervention package, psychosocial interventions, service users and other stakeholders' involvement), clinical research (involvement in clinical research, interest in expanding or incorporating clinical research) domains. Core implementation challenges reported in open-ended question were coded into common categories and included in comparative stratified analyses. Comparative analyses were conducted using Fisher's exact test for categorical variables, and Mann-Whitney *U*-test for continuous variables. All *p*-values given are two-tailed and significance was set to *p* = 0.05. A visual representation of the geographical distribution of the participating services was done using an online application (Maptive; https://www.maptive.com).

## Results

### Service configuration

In total, 24 services from 19 English cities and 16 National Health Service (NHS) trusts participated in the study between the 18th of June and 2nd of September 2021 (Supplementary Figure 1, Supplementary Table 2). This sample represents 22.4% of all targeted early intervention services for psychosis in England, and 33.3% of all corresponding NHS Trusts. Most CHR-P clinical teams (83.3%) were integrated within other mental health services, and only (16.7%) were standalone services (Table 1). There were no hub and spoke services. Over half of the services (66.7%) described their catchment areas as presenting higher-than-average levels of economic deprivation (e.g., unemployment, homelessness), and about half of services as presenting higher-than-average levels of substance use (54.2%), crime and violence (50%), and ethnic minority populations (50%). Regarding workforce composition, services reported an average of 5.7 (SD: 2.8) and a median of 6 professional roles (Table 1): case managers or coordinators were present in 73.7% of services, followed by administrative support roles (68.4%), psychiatrists and CBT practitioners (63.2% each), clinical psychologists (58.9%), and nurses and occupational or vocational workers (42.1% each). The remaining professional roles (e.g., assistant psychologist, mental health support worker, team leader, trainee psychiatrist, social worker, family therapist, and other roles) were present in 26% of services or less (Table 1).

**Table 1:** Service configuration.

**Workforce composition**	**All services, *n* (%)**
*Service model*	*24 (100)*
Integrated	20 (83.33)
Standalone	4 (16.67)
Hub and spoke	0
*Role composition*	*19 (100)*
Case manager or care coordinator	14 (73.68)
Administrative support	13 (68.42)
Psychiatrist	12 (63.16)
CBT therapist	12 (63.16)
Clinical psychologist	11 (58.89)
Nurse	8 (42.11)
Occupational or vocational worker	8 (42.11)
Assistant psychologist	5 (26.32)
Mental health support worker	5 (26.32)
Team leader	5 (26.32)
Trainee psychiatrist	4 (21.05)
Social worker	4 (21.05)
Family therapist	2 (10.53)
Other roles^a^	3 (15.79)
Average number of roles per service (SD)	5.68 (2.81)
Median number of roles per service	6.00

^a^Included roles (services): CBT therapist trainee (1), carer liaison worker (1), clinical lead (1), keyworker (1), research worker (1). CBT, Cognitive-behavioral therapy; CHR-P, Clinical high-risk state for psychosis; NICE, National institute for Health and Care Excellence; SD, Standard deviation. Italics indicate total sample size for each variable across all services.

### Detection of at-risk individuals

Out of 20 services, 15 (75%) reported the presence of regular outreach and service promotion activities (Table 2). Mental health awareness and promotion for professionals (55%) and community organizations (45%), and intensive networking with local or community stakeholders (45%), were the three activities most frequently reported. The full list of outreach activities is available in Table 2 and appear highly heterogeneous. In addition, 42.9% of services reported having a service-dedicated website, 28.6% social media presence, and 38.1% reported no online presence (Table 2). Most services (95.2%) allowed for self-referrals (Table 2). The total yearly (i.e., last 12 months) caseload of CHR-P individuals across 21 services was 693, and the average yearly caseload was 33 (range: 6–115), with a median value of 15 (Table 2). Most services (78.3%) indicated that the caseload of CHR-P service users was not capped at any given time (Table 2). Among the services with a capped caseload, the average maximum number of CHR-P service users was 23 (range: 15–33) (Table 2). In addition, over half of services (65.2%) reported their willingness to expand the caseload of CHR-P service users in the future.

**Table 2:** Detection of at-risk individuals.

**Outreach and service promotion**	**All services, *n* (%)**	**Integrated, *n* (%)**	**Standalone, *n* (%)**	**Statistics^a^**
*Report regular outreach and service promotion activities*	*20 (100)*	*16 (100)*	*4 (100)*	*Fisher's exact test (p-value)*
Yes	15 (75)	11 (68.75)	4 (100)	0.53
Mental health awareness and promotion for professionals	11 (55)	8 (50)	3 (75)	0.59
Intensive networking with local or community stakeholders	9 (45)	5 (31.25)	4 (100)	0.26
Mental health awareness and promotion for community organizations	9 (45)	6 (37.5)	3 (75)	0.29
Training in early psychosis detection skills	7 (35)	3 (18.75)	4 (100)	**0.007**
Psychoeducation workshops aimed at the general population	5 (25)	3 (18.75)	2 (50)	0.25
Mental health awareness and promotion for general population	4 (20)	2 (12.5)	2 (50)	0.16
Print media (e.g., brochures, posters, newsletters)	4 (20)	3 (18.75)	1 (25)	1.00
Media presence (e.g., TV, radio)	2 (10)	2 (12.5)	0 (0)	1.00
Roadshows and presence in public events	2 (10)	1 (6.25)	1 (25)	0.37
Stigma reduction activities/anti-stigma campaigns	2 (10)	1 (6.25)	1 (25)	0.37
Student internships	2 (10)	1 (6.25)	1 (25)	0.37
On-site screening	1 (5)	0 (0)	1 (25)	0.20
No	5 (25)	5 (31.25)	0	.
*Online presence*	*21 (100)*	*17 (100)*	*4 (100)*	*Fisher's exact test (p-value)*
Service-specific website	9 (42.86)	6 (35.29)	3 (75)	0.27
Social media presence	6 (28.57)	3 (17.65)	3 (75)	0.053
None of the above	8 (38.10)	7 (41.18)	1 (25)	1.00
*Availability of self-referral*	*21 (100)*	*17 (100)*	*4 (100)*	*Fisher's exact test (p-value)*
Yes	20 (95.24)	16 (94.12)	4 (100)	1.00
No	1 (4.76)	1 (5.88)	0	.
**Caseload**				
*Is the caseload of CHR-P individuals capped at any given time?*	*23 (100)*	*19 (100)*	*4 (100)*	*Fisher's exact test (p-value)*
Yes	5 (21.7)	5 (26.32)	0	0.54^a^
Average number of CHR-P service users	23 (range: 15-33)	23 (range: 15-33)		.
No	18 (78.3)	14 (73.68)	4 (100)	.
*Intent or interest to increase caseload capacity for CHR-P individuals*	*23 (100)*	*19 (100)*	*4 (100)*	*Fisher's exact test (p-value)*
Yes	15 (65.22)	14 (73.68)	1 (25)	0.10^a^
No	8 (34.78)	5 (26.32)	3 (75)	.
*Yearly caseload^*b*^ of CHR-P or suspected-CHR-P individuals*	*21 services*	*18 services*	*3 services*	*Mann-Whitney U-test*
Total	693	504	189	.
Average	33 (SD: 30.66)	28 (SD: 29.12)	63.00 (SD: 24.88)	*Z = −2.014, p = **0.047***
Range	4–115	4–115	36–85	.
Median	15	15	68	.
*Proportion of CHR-P individuals in yearly caseload^*b*^*	.	*14 services*	*3 services*	.
Total yearly caseload	.	3,063	216	.
Total CHR-P individuals in yearly caseload (percentage over total)	.	441 (14.40)	189 (87.5)	.

CHR-P, Clinical high-risk state for psychosis; SD, Standard deviation.

^a^Comparison of integrated vs. standalone services (there were no hub and spoke services). ^b^Last 12 months. Bold indicates statistically significant values. ^.^, Unavailable or non-applicable. Italics indicate total sample size for each variable across all services, integrated services, and standalone servies.

### Prognostic assessment

Over half of services (56.5%) included service users aged 14–35 years, followed by 14–65 years (13.0%) and 18–35 years (8.7%) (Table 3). Five services (21.7%) accepted users up to 65 years of age. All services accepted users based on the presence of a CHR-P status, as defined by the Comprehensive Assessment of At-Risk Mental States (CAARMS) (95.7%) or Structured Interview for Prodromal Syndromes (SIPS) (4.3%) (Table 3). In addition, 21.7% of services accepted users meeting criteria for DSM-5 Attenuated Psychosis Syndrome. In addition, most services (90%) report the systematic collection of outcomes measures and achievements. The top six more frequent outcomes include service user engagement or satisfaction (95% of services), followed by family or carer satisfaction, general quality of life or wellbeing, and improvement in social functioning (75% each), and psychosis transition rates and improvement in occupational functioning (70% each). The full list of outcomes is available in Table 3.

**Table 3:** Prognostic assessment.

**Intake criteria**	**All services, *n* (%)**	**Psychosis risk assessment**	**All services, *n* (%)**
*Age intake criteria*	*23 (100)*	*Psychosis screening prior to initial assessment*	*22 (100)*
14–35 years	13 (56.52)	Yes	13 (59.1)
14–65 years	3 (13.04)	Telephone	9 (69.23)
18–35 years	2 (8.70)	Face-to-face assessment	6 (46.15)
14–18 years	1 (4.35)	Online	3 (23.08)
15–65 years	1 (4.35)	No	9 (40.9)
16–25 years	1 (4.35)		
16–30 years	1 (4.35)	*Instrument for the assessment of psychosis risk*	*23 (100)*
18–65 years	1 (4.35)	CAARMS	22 (95.65)
*Psychosis risk clinical diagnosis^*a*^*	*23 (100)*	SIPS/SOPS	1 (4.35)
CHR-P status^b^	23 (100)	*Trauma screening tool*	*23 (100)*
DSM-5 attenuated psychosis syndrome	5 (21.74)	Yes	7 (30.4)
Not meeting criteria for PANNS	2 (8.70)	No	16 (69.4)
**Outcome measures**	**All services**, ***n*** **(%)**	**Integrated**, ***n*** **(%)**	**Standalone**, ***n*** **(%)**	**Statistics** ^c^
*Are outcomes measures and achievements systematically reviewed?*	*20 (100)*	*16 (100)*	*4 (100)*	*Fisher's exact test (p-value)*
Yes	18 (90)	14 (87.5)	4 (100)	1.00
No	2 (10)	2 (12.5)	0	.
*Regularly evaluated outcomes for CHR-P individuals*	*20 (100)*	*16 (100)*	*4 (100)*	*Fisher's exact test (p-value)*
Service user engagement or satisfaction with service	19 (95)	15 (93.75)	4 (100)	1.00
Family or carer satisfaction with service	15 (75)	11 (68.75)	4 (100)	0.53
General quality of life or wellbeing	15 (75)	12 (75)	3 (75)	1.00
Improvement in social functioning	15 (75)	13 (81.25)	2 (50)	0.55
Psychosis transition rates	14 (70)	10 (62.5)	4 (100)	0.27
Improvement in occupational functioning	14 (70)	12 (75)	2 (50)	0.25
Symptomatic persistence, improvement and remission	13 (65)	10 (62.5)	3 (75)	1.00
Improvement in comorbid mental health conditions	13 (65)	11 (68.75)	2 (50)	0.59
Number of users entering employment or education	11 (55)	8 (50)	3 (75)	0.59
Time to receiving treatment	10 (50)	8 (50)	2 (50)	1.00
Number of referrals to service	7 (35)	6 (37.5)	1 (25)	1.00
Hospitalization	6 (30)	5 (31.25)	1 (25)	1.00
Physical health	6 (30)	6 (37.5)	0 (0)	0.27
Other outcomes regarding service operation	4 (20)	4 (25)	0 (0)	0.54

CAARMS, Comprehensive Assessment of At-Risk Mental States; CHR-P, Clinical high-risk state for psychosis; PANNS, Positive and Negative Syndrome Scale; SIPS, Structured Interview for Prodromal Syndromes; SOPS, Scale of Prodromal Symptoms.

^a^Not mutually exclusive.

^b^CAARMS, SIPS/SOPS.

^c^Comparison of integrated vs. standalone services (there were no hub and spoke services). Italics indicate total sample size for each variable across all services, integrated services, and standalone servies.

### Clinical care

Duration of service provision was of 24 months across most services (33.3%), followed by 36 months (22.2%), 12 months (22.2%), 18 months (16.7%), and 6 months (5.6%) (Table 4). Regarding NICE-compliant psychosocial interventions, 65% of services report the provision (as yes/no answer) of some clinical monitoring plus NICE-standard CBT plus NICE-standard family therapy, 30% of clinical monitoring plus NICE-standard CBT, and 5% clinical monitoring plus NICE-standard family therapy. The full list of offered psychosocial interventions is available in Table 4. However, among 10 services with available data, the actual yearly proportion of CHR-P individuals receiving CBT was 66.5%; among 12 services, only 4.9% of the yearly caseload received family-oriented interventions (Table 4). Approximately half of CHR-P services (47.4%) involved service users, 26.3% family members or carers, and 15.8% past beneficiaries in various service operations or activities (Table 4), including service promotion and development (40% of services each), service evaluation and staff recruitment (30% each), service delivery (25%), and research planning or promotion (10%) (Table 4).

**Table 4:** Clinical care.

**Service provision**	**All services, *n* (%)**	**Integrated, *n* (%)**	**Standalone, *n* (%)**	**Statistics^a^**
*Duration of service provision (including monitoring)*	*18 (100)*	*14 (100)*	*4 (100)*	*Mann-Whitney U-test*
36 months	4 (22.22)	4 (28.57)	0	.
24 months	6 (33.33)	2 (14.29)	4 (100)	.
18 months	3 (16.67)	3 (21.43)	0	.
12 months	4 (22.22)	4 (28.57)	0	.
6 months	1 (5.56)	1 (7.14)	0	.
Average (SD)	22 (9.43)	21.43 (10.71)	24 (0)	*Z = −0.877, p = 0.44^*a*^*
*NICE-compliant intervention packages*	*20 (100)*	*16 (100)*	*4 (100)*	*Fisher's exact test (p-value)*
Monitoring of mental state + CBT + family therapy	13 (65)	10 (62.5)	3 (75)	1.00
Monitoring of mental state + CBT	6 (30)	5 (31.25)	1 (25)	1.00
Monitoring of mental state + family therapy	1 (5)	1 (6.25)	0	1.00
*Offered individual psychosocial interventions*	*20 (100)*	*16 (100)*	*4 (100)*	*Fisher's exact test (p-value)*
Monitoring of mental state	20 (100)	15 (93.75)	4 (100)	1.00
Psychoeducation (service-users)	19 (95)	15 (93.75)	4 (100)	1.00
NICE-standard CBT for psychosis	19 (95)	15 (93.75)	4 (100)	1.00
CBT-informed interventions	18 (90)	14 (87.5)	4 (100)	1.00
Psychoeducation (family/carers)	16 (80)	12 (75)	4 (100)	0.54
Crisis work	16 (80)	13 (81.25)	3 (75)	1.00
Other individual psychotherapy	14 (70)	10 (62.5)	4 (100)	0.27
NICE-standard family therapy	14 (70)	11 (68.75)	3 (75)	1.00
Occupational or employment support	14 (70)	11 (68.75)	3 (75)	1.00
Housing or benefits support	14 (70)	10 (62.5)	4 (100)	0.27
Recovery-oriented case management	13 (65)	9 (56.25)	4 (100)	0.25
Family work and support	12 (60)	9 (56.25)	3 (75)	0.62
Social functioning (e.g., social skills training)	10 (50)	8 (50)	2 (50)	1.00
Educational/academic support	10 (50)	8 (50)	2 (50)	1.00
Mental Health Act work	8 (40)	6 (37.5)	2 (50)	1.00
Substance misuse work	6 (30)	5 (31.25)	1 (25)	1.00
Recreational group activities	6 (30)	4 (25)	2 (50)	0.55
Daily living skills training	5 (25)	5 (31.25)	0 (0)	0.53
Peer-support	4 (20)	4 (25)	0 (0)	0.54
Group psychotherapy	2 (10)	0 (0)	2 (50)	**0.032**
**Provision of NICE-standard interventions in yearly**^b^ **caseload**	***n*** **(%)**	**Yearly caseload of CHR-P individuals, n**	***Yearly CHR-P individuals receiving interventions, n*** **(%)**	
*All services*	*20 (100)*	.	.	
NICE-standard CBT offered	9 (45)	261	181 (69.35)	
NICE-standard CBT offered but data unavailable	10 (50)	.	.	
NICE-standard CBT not offered	1 (5)	11	.	
Total services with available data	10 (50)	272	181 (66.54)	
**Service provision**	**All services, *n* (%)**	**Integrated, *n* (%)**	**Standalone, *n* (%)**	**Statistics^a^**
*All services*	*20 (100)*	.	.	
NICE-standard family therapy offered	6 (30)	140	16 (11.43)	
NICE-standard family therapy offered but data unavailable	8 (40)	.	.	
NICE-standard family therapy not offered	6 (30)	187	.	
Total services with available data	12 (60)	327	16 (4.89)	
**Service users and other stakeholders' involvement**	**All services**, ***n*** **(%)**	**Integrated**, ***n*** **(%)**	**Standalone**, ***n*** **(%)**	**Statistics** ^a^
*Service users and other stakeholders' involvement*	*19 (100)*	*15 (100)*	*4 (100)*	*Fisher's exact test (p-value)*
Service users	9 (47.37)	6 (40)	3 (75)	0.30
Family members or carers	5 (26.32)	4 (26.67)	1 (25)	1.00
Past beneficiaries	3 (15.79)	1 (6.67)	2 (50)	0.10
Any of the above	10 (52.63)	7 (46.67)	3 (75)	0.58
Limited to FEP	1 (5.26)	1 (6.67*)*	0	.
*Activities incorporating service user involvement*	*20 (100)*	*16 (100)*	*4 (100)*	*Fisher's exact test (p-value)*
Service promotion	8 (40)	5 (40)	3 (75)	0.26
Development of services or products	8 (40)	6 (37.5)	2 (50)	1.00
Service evaluation	6 (30)	3 (18.75)	3 (75)	0.061
Staff recruitment	6 (30)	4 (25)	2 (50)	0.55
Service delivery	5 (25)	3 (18.75)	2 (50)	0.25
Research planning or promotion	3 (10)	1 (6.25)	2 (50)	0.088

CBT, Cognitive behavioral therapy; CHR-P, Clinical high-risk state for psychosis; FEP, First-episode of psychosis; NICE, National institute for Health and Care Excellence; SD, Standard deviation.

^a^Comparison of integrated vs. standalone services (there were no hub and spoke services). Bold indicates statistically significant values.

^b^Last 12 month. ^.^, Unavailable or non-applicable. Italics indicate total sample size for each variable across all services, integrated services, and standalone servies.

### Clinical research

Approximately half of services (47.8%) were involved in clinical research, and most (91.3%) were in principle interested in expanding or incorporating clinical research (Supplementary Table 3). Research into evidence-based preventive interventions was felt particularly needed (55% of services). Other areas considered in need of further research included addressing long-term outcomes of CHR-P service users and the identification of users in need of extended care (20% of services), improving accuracy of psychosis risk assessments (20%) and access and engagement with services (15%), and research into different service models (20%). The full list of areas considered in need of further research is available in Supplementary Table 3.

### Implementation challenges

This section summarizes the most frequent implementation challenges reported by CHR-P services across core implementation domains (full list available in Supplementary Table 4). In terms of service configuration, the recruitment of specialized roles (66.7% of services), lack of dedicate funding for the CHR-P pathway (58.3%), high staff turnover (25%) and insufficient funding for staff training (25%) were the four most common implementation challenges. Regarding detection of at-risk individuals, limited resources were key challenges for both expanding current service outreach activities (50%) and increasing the current number of referrals (33.3%). Regarding the prognostic assessment domain, the need for training opportunities in psychosis risk assessment was reported by 37% of services, followed by the need to enhance the accuracy of current psychosis risk assessments (20.8%). Common implementation challenges related to clinical care included the need for training in clinical intervention skills (41.7%), low engagement with virtual interventions and digital poverty among users (33.3%), higher than recommended caseloads (25%), and limited resources to meet the national preventive targets (25%). Finally, among 12 services not involved in clinical research, implementation challenges included insufficient time or personnel (83.3%), insufficient funding (75%), insufficient training or skills (66.7%), and research not being considered a priority (41.7%).

### Comparative analysis of integrated vs. standalone services

This section summarizes comparative analyses between integrated and standalone services. In terms of detection of at-risk individuals (Table 2), there were no differences regarding intensity of regular outreach and service promotional activities. Similarly, no significant differences were found for online presence and availability of self-referrals between. However, standalone services reported a significantly higher yearly caseload of CHR-P individuals than integrated services (average: 63 vs. 28, *p* = 0.047) (Table 2). Notably, among integrated services with available data (*n* = 14), CHR-P individuals represented only 14.4% of all yearly caseload (vs. 87.5% in 3 standalone services), because these teams were more frequently providing care to FEP individuals. In terms of regularly evaluated outcomes, no statistical differences were found between integrated and standalone services (Table 2). Regarding interventions and provision of care (Table 4), there were no differences with respect to the provision of NICE-compliant interventions packages. In terms of specific psychosocial interventions and service users and other stakeholders' involvement activities, there was only a higher provision of group psychotherapy (*p* = 0.032) across standalone services. Standalone services were also more likely to conduct clinical research than integrated services (*p* = 0.037). Finally, integrated services were more likely to report lack of dedicated budget as a core implementation challenge (*p* = 0.020) as well as to report more severe implementation challenges.

## Discussion

To our best knowledge this analysis represents one of the largest national audits of CHR-P services worldwide. Research into established CHR-P services can help inform decision making for current and future CHR-P services in the United Kingdom and overseas. This is a particularly pressing need as various regions prepare to extend coverage of early psychosis services, such as Scotland's Mental Health Strategy 2017–2027 (49). As such, in this section we offer a discussion and recommendations for CHR-P services (summarised in Supplementary Table 5).

In terms of service configuration, this report provides an in-depth overview of 24 CHR-P services across 19 English cities and 16 NHS trusts, representing 22.4% of all targeted early intervention services for psychosis and 33.3% of NHS Trusts in England. In 2020/2021, the coverage of CHR-P within 154 early intervention services was limited to 41–68% of services across all age groups (50). One core finding in this domain is that most (83%) CHR-P services in our sample were integrated as an adjunct component of broader mental health teams, particularly FEP services. A similar proportion of integrated vs. standalone CHR-P services (80.4 vs. 19.6%, respectively) can also be found globally (31). Integrating CHR-P teams in other mental health services is likely to penalize the provision of preventive care to CHR-P individuals as time and clinical resources are directed toward the most unwell users (33). These observations have been fully corroborated by our comparative analysis of core domains across service configuration, as detailed below. Importantly, most CHR-P services have been implemented in areas with higher levels of socioeconomic deprivation, highlighting a window of opportunities for impacting core social factors determining the onset of psychosis (13). In a recent review study, we highlighted that CHR-P services have the potential to deliver several public-health preventive approaches for the local community that target these factors (47). Regarding role composition, CHR-P services require the synergy of a diverse team of professionals responsible for conducting specialized assessments, delivering psychosocial and pharmacological treatments, implementing outreach and service promotion activities and, potentially, coordinating and conducting clinical research (31–33). CHR-P services were therefore multidisciplinary, with a median of 6 distinct roles per service. However, this also translated in difficulties for the recruitment of specialized roles, a core implementation challenge affecting CHR-P services regardless of their service model. In addition, implementation challenges relating to this domain, particularly among integrated services, included the lack of dedicated funding for the CHR-P pathway that affect human resources and training. Overall, a stand-alone service model with dedicated funding and resources appears as the preferable option as indicated by evidence from well-established services (32, 33). A standalone model allows, among other, for more effective outreach and service-user intake, and overall quality of clinical care to CHR-P individuals. However, securing funding from commissioners is challenging and partly dependant on socioeconomic and political factors on a local and national level. New or developing services can benefit from available guidelines developed by more experiences services when producing their business case (51). On the other hand, alternative service models, such as integrated or ‘hub and spoke', are more prevalent in developing countries with incipient implementation of early psychosis services and limited funding opportunities (52).

Detecting at-risk individuals is the first rate-limiting step for the large-scale implementation of the CHR-P paradigm (10), which is then followed by assessing psychosis risk (53) and delivering indicated phase specific evidence-based interventions (54). Detection of CHR-P individuals is typically non-systematic (55), and dependant on the availability of means and resources within each service. For example, we clearly observed heterogeneous outreach campaigns that were not standardized across the nation. This resulted in a highly variable recruitment capacity, with a median yearly caseload of only 15 individuals per CHR-P service and a total national yearly recruitment capacity of 693 CHR-P individuals. Assuming a yearly number of 6,833 new cases of psychotic disorders in England (2022 estimate for ages 16–35, https://www.psymaptic.org/), and a likelihood of developing FEP from a CHR-P of 20% (at 2 years) (22) it is evident that to date, the preventive capacity of the CHR-P paradigm is still largely unexploited in England, with numerous young people putatively presenting with CHR-P features remaining undetected. Implementation challenges relating to this domain were corroborated by the observation that over half of services in our sample reported lack of recruitment capacity due to high demand or dedicated funding. Limited recruitment capacity of CHR-P services across England might constitute a core barrier for the identification of subjects who might benefit from specialized support. Inefficient recruitment strategies result in only 5% (56) to 12% (57) of users receiving care for a FEP having previously been offered support during the CHR-P stage. In the future, novel automatic detection methods based on individualized transdiagnostic (58–60) or poly-environmental (61, 62) risk calculators, as well as e-detection strategies aimed at the general population (63), can help narrow the detection gap (53). In comparative analyses, the yearly caseload of CHR-P individuals in standalone services doubled that of integrated services. This, coupled with CHR-P individuals representing a minority of people receiving direct clinical care within integrated services, indicates that standalone services are more successful at detecting at-risk individuals. In addition, it highlights how preventive efforts risk becoming diluted among CHR-P services where the clinical care is embedded with other patient populations. Until novel solutions for the detection of at-risk individuals become suitable for real-world implementation, recruitment strategies should be resource-efficient and ensure adequate levels of pre-test risk enrichment (55, 64). Outreach can include a combination of active and passive strategies, including internet-based platforms with self-referral options. For active outreach, collaborative relationships should be established with key local stakeholders, with a priority given to clinical services working with adolescents and 379 young adults, given the higher pre-test psychosis risk among clinical samples (10).

Regarding prognostic assessment, our results are consistent with previous studies reporting 13–15 to 30–35 years as the most frequent age inclusion criteria for CHR-P service in England (45) and globally (31). In our sample, however, 21.7% of CHR-P services provided coverage for people up to 65 years of age, a higher proportion than previously reported for global CHR-P services (31), likely reflecting recent national guidance requiring lifespan early interventions for psychosis (46). However, while the 15–35 age range corresponds with the epidemiological period of increased risk for psychosis onset, age older than 35 acts as a protective factor (13). Therefore, extending provision of CHR-P care to people over 35 (46, 50) is not supported by epidemiological evidence. Furthermore, CHR-P assessment instruments have been validated for the 14–35 age range (65). Therefore, no valid CHR-P assessment tools for older age groups exist. Unmet training needs in assessment skills, and the need to enhance the accuracy of current psychosis risk assessment instruments, emerged as core implementation challenges in this domain. On a commissioning-level, the implementation of virtual learning programmes can be a far-reaching and cost-efficient solution for training needs in psychosis risk assessment. Another interesting finding is that most CHR-P services indicated some systematic collection of clinical outcomes, although there was no consistency in the way outcomes were selected. Limited resources for collecting and processing outcomes, coupled with the need for shared guidelines for outreaching and better integration with current data capture system, were also common barriers. For example, standalone CHR-P services that leverage electronic health records have been successful in completing complex and long-term automatic follow-up of at-risk individuals, addressing real-world outcomes and clinical needs (23). In addition, regional networks of CHR-P services (33) can facilitate the harmonization of core outcomes through collaborative efforts. In comparative analysis, we observed no significant differences for regularly evaluated outcomes. However, almost 40% of the integrated services did not report systematically monitoring transition risk, a core outcome within the CHR-P and psychosis prevention paradigm (10). Overall, systematic collection of clinical outcomes is key to monitoring service performance (51). Key clinical outcomes for CHR-P include not only transition risk, but also measures of overall functioning and service user satisfaction (31). Moreover, resource-constrained services should prioritise the 14–35 age range, the period of highest psychosis risk, for maximum impact and efficiency.

In terms of clinical care, reported duration of service provision in our sample was of 18 months or less (44.5% of services), 24 months (33.3%), or 36 months (22.2%). This duration of care often does not seem sufficient to adequately cover the period of increased transition risk, which continues increasing from 20% at 2 years to 29% at 4 years to 35% at 10 years (22). Notably, a rebound increase in the risk of psychosis has been observed soon after the discharge from CHR-P teams at 2 years (22). Extending standard care beyond the 2-year period is further supported by the presence of other-than-transition long-term poor mental health outcomes among CHR-P individuals, including admission into a mental health hospital, initiation of psychotropic treatment, and increased risk of premature death (23). Furthermore, transition risk varies substantially across the different CHR-P clinical subgroups (66), with BLIPS individuals showing the highest risk and unmet needs (19–21, 66–70). Consequently, a revised CHR-P paradigm based on the stratification between CHR-P subgroups, might be a more efficient alternative (6, 71). We also found that 65% of CHR-P services reported offering a NICE-compliant interventions package encompassing regular monitoring of mental state, NICE-standard CBT, and NICE-standard family therapy. However, this finding was based on a categorical response (yes/not) that did not address the extent and granularity of preventive care implemented in each site. When this was investigated, only 65.5 and 4.9% of CHR-P individuals were offered CBT or family interventions, respectively. This clearly represents a suboptimal threshold of implementation for preventive care. Concernedly, several sites did not have data on preventive care offered and more than half of those namely offering CBT or family therapy were unable to provide the specific number of service users receiving these interventions. These findings might reflect the operational challenges that CHR-P services face when effectively delivering treatments *vis-à-vis* limited capacity and implementation challenges, as well as the limited efficacy of the recommended psychological interventions (7, 54, 72–75). Unmet training needs in clinical intervention skills were a core implementation challenge. Low service user engagement with virtual interventions in the context of the COVID-19 outbreak, and limited capacity alongside higher than recommended caseloads or increasing demand were also reported. These obstacles combine with challenges related to budget and workforce, representing a barrier for the widespread compliance with national commissioning guidance (46). Alongside NICE-recommended interventions, CHR-P services reported the provision of several needs-based interventions related to occupational, practical, and social requirements. Notably, standalone services in our sample were more likely to offer group psychotherapy (*p* = 0.032). Based on the most up-to-date clinical and epidemiological evidence, the European College of Neuropsychopharmacology Network on the Prevention of Mental Disorders and Mental Health Promotion has suggested a cautious approach centered on need-based interventions and psychotherapy (CBT or integrated psychological interventions) titrated on the specific risk profile across clinical subgroups and the individual's values and preferences (10). However, the provision of needs-based interventions is inconsistent and not widespread across CHR-P services. In addition, as indicated by the limited coverage of CBT and family therapy, complex psychosocial interventions can fail to be delivered to many service users. Overall, standard duration of clinical care and monitoring should extend to a recommended minimum of 3 years, to cover the period of increased transition risk (22), and risk of other severe real-world outcomes (23). Finally, a more efficient use of clinical resources can be achieved by titrating interventions based on individual risk profiles (i.e. transition risk according to CHR-P clinical subgroups, symptoms severity, and functional impairment) and individual preferences.

In terms of clinical research, standalone CHR-P services are most successful, being more likely to participate in clinical research than integrated services (*p* = 0.037). In fact, only one in three integrated services reported involvement in clinical research (vs. all standalone services). As with previous domains, lack of capacity and funding were core barriers among integrated services for conducting clinical research in psychosis prevention. In addition, research is often not considered a priority. This is unsurprising, as limited resources in integrated services need to be distributed across preventive efforts and the provision of care to more urgent cases. Standalone services, on the other hand, can focalize resources into psychosis prevention-related projects. This is illustrated by the OASIS service in South London, which has attracted £50 million grant income during the 2010–2022 period and produced high-impact research outputs, as reflected by almost 6,000 OASIS-related citations by March 2020 (32). Both within the United Kingdom (33) and overseas (35, 36), regional or national networks of CHR-P and FEP services continue to be established. The creation or expansion of regional networks can act as a gateway for emerging services into research activities, by leveraging resources, research initiatives, and expertise of better-established ones. Finally, the harmonisation of clinical outcome measures across CHR-P services would be an important step towards improved research.

One limitation is the use of a convenience sampling strategy. Therefore, we do not claim our results to be representative of all CHR-P services worldwide. Nonetheless, we extend a previous report of a London-based network of CHR-P services (33) to incorporate services in the South and North of the England. Also, the reduced number of standalone services in our sample resulted in small statistical power. As such, our comparative analyses should be considered exploratory, requiring future replication through higher-powered studies. Finally, the period for data collection overlapped with the COVID-19 pandemic, which often disrupted normal operations of NHS secondary mental health services (76, 77). As a result, some of our results might not accurately reflect the normal operations of CHR-P services during the pre-pandemic period.

## Conclusion

While implementation of CHR-P services is observed in several parts of England, only standalone teams appear successful at detection of at-risk individuals. Compliance with NICE-prescribed interventions is limited across CHR-P services and unmet needs emerge for national training and investments.

## Data availability statement

The original contributions presented in the study are included in the article/Supplementary material, further inquiries can be directed to the corresponding author.

## Author contributions

AE contributed to study design, data collection, and manuscript writing and editing. PF-P contributed to study design and manuscript writing and editing. SM-A contributed to data collection and manuscript writing and editing. TS, AD, UP, and PM contributed to manuscript review and editing. All authors contributed to the article and approved the submitted version.
