# Supplementary material for: Mapping the implementation and challenges of clinical services for psychosis prevention in England
Source: Front Psychiatry. 2023 Jan 3;13:945505. doi: 10.3389/fpsyt.2022.945505 (PMC9844094; doi:10.3389/fpsyt.2022.945505)
Supplement: Supplementary file 1 [file Table_1.DOCX]

# **eTable 1.** Structure of audit

| Domain | Variables |
| --- | --- |
| Service configuration | - Service name, city, and creation date - Service model - Catchment area characteristics - Workforce composition - Implementation challenges |
| Detection of at-risk individuals | - Outreach and service promotion activities - Online presence - Availability of self-referral - Capped caseload of CHR-P individuals - Intent to increase caseload of CHR-P individuals - Annualised caseload of CHR-P individuals - Implementation challenges |
| Prognostic assessment | - Age inclusion criteria - Psychosis risk clinical diagnosis - Additional exclusion criteria - Psychosis screening prior to initial assessment - Instrument for the assessment of psychosis risk - Trauma screening - Systematic review of outcomes and achievements - Regularly evaluated outcomes for CHR-P individuals - Implementation challenges |
| Clinical care | - Duration of service provision - Psychosocial interventions currently offered - Involvement of service users, family members or carers, and past beneficiaries - Activities incorporating service user involvement - Implementation challenges |
| Clinical research | - Clinical research conducted at the service - Interest in expanding or incorporating research in the future - Pressing areas in need of research and innovation in psychosis prevention - Implementation challenges |

Legend: CHR-P, Clinical high-risk state for psychosis.
