# Supplementary material for: Mapping the implementation and challenges of clinical services for psychosis prevention in England
Source: Front Psychiatry. 2023 Jan 3;13:945505. doi: 10.3389/fpsyt.2022.945505 (PMC9844094; doi:10.3389/fpsyt.2022.945505)
Supplement: Supplementary file 2 [file Table_2.DOCX]

# **eTable 2.** Clinical services included in the audit

| Service name | City | Creation year |
| --- | --- | --- |
| Aspire Early Intervention Psychosis – Community Links | Leeds | 2018 |
| Barnsley Early Intervention Team | Barnsley | 2018 |
| Banes Early Interventions Team | Bath | 2016 |
| Bradford and Airedale Early Intervention Service | Bradford | 2018 |
| Bradford, Airedale and Craven EIP CAMHS | Bradford | 2016 |
| Bury Early Intervention Team | Manchester | 2016 |
| Cambridgeshire and Peterborough Assessing, Managing and Enhancing Outcomes (CAMEO) | Cambridge | NA |
| CENTRAL ARMS Service | Newcastle | 2020 |
| East Cheshire Early Intervention Team | Winsford | 2016 |
| City and Hackney Early Detection Services (HEADS UP) | London | 2015 |
| Doncaster EIP Team – ARMS Service (ARMSp) | Doncaster | 2018 |
| Early Intervention Team Lincolnshire | Lincoln | 2017 |
| Mersey Care Early Intervention in Psychosis Service | Liverpool | 2005 |
| Newham Early Intervention Service (NEIS) | London | 2006 |
| North Kirklees Insight Team | Dewsbury | 2017 |
| Oldham Early Intervention Team | Oldham | 2003 |
| Outreach and Support in South London (OASIS) Lewisham and Croydon | London | 2016 |
| Outreach and Support in South London (OASIS) Southwark and Lambeth | London | 2001 |
| Oxford Health NHS Foundation Trust Early Intervention Service | Oxford | NA |
| Rotherham Early Intervention (RDASH) | Rotherham | NA |
| Worthing Early Intervention in Psychosis Service | Worthing | NA |
| Tower Hamlets Early Detection Service (THEDS) | London | 2009 |
| Wakefield Early Intervention Team | Wakefield | NA |
| Widnes Early Intervention in Psychosis Service | Widnes | NA |

Legend: ARMS, At-Risk Mental State for Psychosis; CAMHS, Child and Adolescent Mental Health Services; EIP, Early Intervention in Psychosis; EIS, Early Intervention Service; NHS, National Health Service.
