# Supplementary material for: Mapping the implementation and challenges of clinical services for psychosis prevention in England
Source: Front Psychiatry. 2023 Jan 3;13:945505. doi: 10.3389/fpsyt.2022.945505 (PMC9844094; doi:10.3389/fpsyt.2022.945505)
Supplement: Supplementary file 3 [file Table_3.DOCX]

# **eTable 3.** Clinical research

| **Clinical research at the service** | **All services, n (%)** | **Integrated, n (%)** | **Standalone, n (%)** | **Statistics^a^** |
| --- | --- | --- | --- | --- |
| *Clinical research conducted at the service*  Yes  No  *Interested in expanding or incorporating research at the service*  Yes  No  *Main areas considered in need of research in psychosis prevention*  Preventive interventions  Long-term outcomes and the identification need of extended care  Accuracy of psychosis risk assessments  Different service models  Improving access and engagement with services  Psychosis risk and care of people aged 35 or over  e-health solutions or technology  Trauma and psychosis  Psychosis risk and emerging personality disorders | *23 (100)*  11 (47.82)  12 (52.17)  *23 (100)*  21 (91.30)  2 (8.70)  *20 (100)*  11 (55)  4 (20)  4 (20)  4 (20)  3 (15)  2 (10)  2 (10)  2 (10)  1 (5) | *19 (100)*  7 (36.84)  12 (63.16)  *19 (100)*  18 (94.74)  1 (5.26)  .  .  .  .  .  .  .  .  .  . | *4 (100)*  4 (100)  0  *4 (100)*  3 (75)  1 (25)  .  .  .  .  .  .  .  .  .  . | *Fisher’s exact test (p value)*  **0.037**  .  *Fisher’s exact test (p value)*  0.32  .  .  .  .  .  .  .  .  .  .  . |

^a^Comparison of integrated vs standalone services (there were no hub and spoke services). Bold indicates statistically significant values.
