# Supplementary material for: Mapping the implementation and challenges of clinical services for psychosis prevention in England
Source: Front Psychiatry. 2023 Jan 3;13:945505. doi: 10.3389/fpsyt.2022.945505 (PMC9844094; doi:10.3389/fpsyt.2022.945505)
Supplement: Supplementary file 4 [file Table_4.DOCX]

# **eTable 4.** Implementation challenges^a^

| **Implementation challenges** | **All services, n (%)** | **Integrated, n (%)** | **Standalone, n (%)** | **Statistics^b^** |
| --- | --- | --- | --- | --- |
| *Service configuration*  Recruitment or access to specialised roles^c^  Lack of dedicate budget for CHR-P pathway  Staff retention or turnover  Insufficient funding for staff training  Limited access or availability of training opportunities  *Detection of at-risk individuals*  Limited resources to expand service outreach activities  Limited resources to accept an increase in referrals  Reduction of outreach activities due to COVID-19 pandemic  Improve training and communication with referral sources  *Prognostic assessment*  Training in psychosis risk assessment  Need to enhance the accuracy of current psychosis risk assessments  Limited capacity or resources for collection of outcomes data  Shared standardised guidelines for collection of outcomes data  Assessment of complex or high-comorbid cases  Reduce assessment waiting times  Better integration of outcomes data with current record systems  *Clinical care*  Training in clinical intervention skills or CHR-P specific interventions^d^  Low engagement with virtual interventions and digital poverty among users  Higher than recommended caseload or increasing demand  Limited resources to meet national preventive targets  Inadequate infrastructure or systems for telemedicine solutions  Limited funding and capacity for service user involvement activities  Expansion of current offer of psychosocial interventions  Increased complexity of clinical presentation  *Clinical research^e^*  Insufficient time or personnel  Insufficient funding  Insufficient training or skills  Not considered a priority | *24 (100)*  16 (66.67)  14 (58.33)  6 (25)  6 (25)  5 (20.83)  *24 (100)*  12 (50)  8 (33.33)  5 (20.83)  4 (16.67)  *24 (100)*  9 (37)  5 (20.83)  4 (16.67)  4 (16.67)  3 (12.50)  2 (8.33)  2 (8.33)  *24 (100)*  10 (41.67)  8 (33.33)  6 (25)  6 (25)  5 (20.83)  5 (20.83)  5 (20.83)  3 (12.5)  *12 (100)*  10 (83.33)  9 (75)  8 (66.67)  5 (41.67) | *20 (100)*  14 (70)  14 (70)  5 (25)  6 (30)  4 (20)  *20 (100)*  11 (55)  7 (35)  4 (20)  4 (20)  *20 (100)*  8 (40)  4 (20)  3 (15)  3 (15)  3 (15)  2 (10)  2 (10)  *20 (100)*  7 (35)  6 (30)  5 (25)  6 (30)  4 (20)  5 (25)  5 (25)  1 (5)  *12 (100)*  10 (83.33)  9 (75)  8 (66.67)  5 (41.67) | *4 (100)*  2 (50)  0  1 (25)  0  1 (25)  *4 (100)*  1 (25)  1 (25)  1 (25)  0  *4 (100)*  1 (25)  1 (25)  1 (25)  1 (25)  0  0  0  *4 (100)*  3 (75)  2 (50)  1 (25)  0  1 (25)  0  0  2 (50)  .  .  .  .  . | *Fisher’s exact test (p value)*  0.58  **0.020**  1.00  0.54  1.00  *Fisher’s exact test (p value)*  0.59  1.00  1.00  1.00  *Fisher’s exact test (p value)*  1.00  1.00  0.54  0.54  1.00  1.00  1.00  *Fisher’s exact test (p value)*  0.27  0.58  1.00  0.54  1.00  0.54  0.54  0.061  .  .  .  .  . |

CHR-P, Clinical high-risk state for psychosis. ^a^Only themes with at least two mentions are reported in the table. ^b^Comparison of integrated vs standalone services (there were no hub and spoke services). ^c^Role-specific needs included: Cognitive-behavioural therapy (CBT) therapist (4 services), clinical psychologist (3 services), family therapist (3 services), peer support or youth worker (3 services), vocational support workers (2 services), psychiatrists (1 service), nutritionist or trainer (1 service). ^d^Clinical training specific needs included: trauma work (3 services), CBT (2 services), clinical work with young service users (2 services), family interventions (1 service), dialectic-behavioural therapy (1 service), eye movement desensitization and reprocessing therapy (1 service). ^e^Only services not involved in clinical research. Bold indicates statistically significant values.
