# Supplementary material for: Mapping the implementation and challenges of clinical services for psychosis prevention in England
Source: Front Psychiatry. 2023 Jan 3;13:945505. doi: 10.3389/fpsyt.2022.945505 (PMC9844094; doi:10.3389/fpsyt.2022.945505)
Supplement: Supplementary file 5 [file Table_5.docx]

**eTable 5.** Ten recommendations for real-world implementation of CHR-P services^a^

| **Service configuration** | |
| --- | --- |
| 1 | Prioritise a stand-alone community service model as the preferable option for maximum efficacy and efficiency. |
| 2 | Take advantage of available guidelines developed by more experiences services when developing a business plan. |
| **Detection of at-risk individuals** | |
| 3 | Identify, contact, and establish close collaborative relationships with key local stakeholders for the identification and referral of individuals in need of specialised assessment and care. |
| 4 | Prioritise healthcare agents as key partners to maximise levels of pre-test psychosis risk (risk enrichment) among individuals referred to the clinical service. |
| **Prognostic assessments** | |
| 5 | Monitor service performance through the systematic collection of key outcomes. |
| 6 | Prioritise the 14-35 age range, the period of highest psychosis risk, for maximum impact and resource efficiency. |
| **Clinical care** | |
| 7 | Clinical care and monitoring should extend to a recommended minimum of 3 years to cover the period of increased transition risk, and risk of other severe real-world outcomes. |
| 8 | Titrate interventions based on individual risk profiles (i.e. CHR-P clinical subgroups^b^, symptoms severity, and functional impairment) and individual preferences. |
| **Clinical research** | |
| 9 | Facilitate research in emerging CHR-P services through the creation or expansion of regional networks. |
| 10 | Harmonise clinical outcome measures across CHR-P services to improve quality of research. |

^a^Adapted from our previous publication: Salazar de Pablo G, Estradé A, Cutroni M, Andlauer O, Fusar-Poli P. Establishing a clinical service to prevent psychosis: what, how and when? Systematic review. *Transl Psychiatry*. 2021;11(1):43. DOI: 10.1038/s41398-020-01165-x.  ^b^CHR-P subgroups transition risk profile BLIPS > APS > GRD.
