# Supplementary material for: Mapping the implementation and challenges of clinical services for psychosis prevention in England
Source: Front Psychiatry. 2023 Jan 3;13:945505. doi: 10.3389/fpsyt.2022.945505 (PMC9844094; doi:10.3389/fpsyt.2022.945505)
Supplement: Supplementary file 6 [file Image_1.PDF]

eFigure 1. Geographical distribution of early intervention services included in the audit

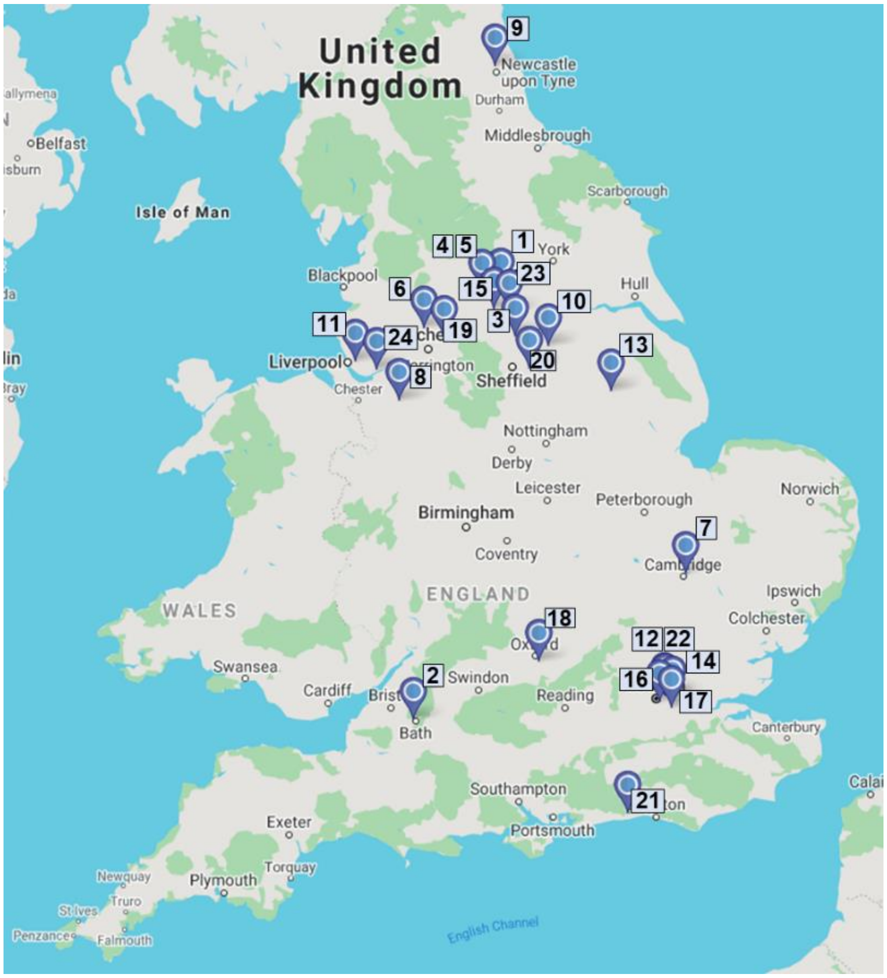

Legend: 1, Aspire Early Intervention Psychosis – Community Links; 2, Banes Early Interventions Team; 3, Barnsley Early Intervention Team; 4, Bradford and Airedale Early Intervention Team; 5, Bradford, Airedale and Craven EIP CAMHS; 6, Bury Early Intervention Team; 7, Cambridgeshire and Peterborough Assessing, Managing and Enhancing Outcomes (CAMEO); 8, East Cheshire Early Intervention Team; 9, CENTRAL At-Risk Mental State (ARMS) Service; 10, Doncaster Early Intervention in Psychosis Team – At Risk Mental State for Psychosis Service (ARMSp); 11, Mersey Care Early Intervention in Psychosis Service; 12, City and Hackney Early Detection Services (HEADS UP); 13, Early Intervention Team Lincolnshire; 14, Newham Early Intervention Service (NEIS); 15, North Kirklees Insight Team; 16, Outreach and Support in South London (OASIS), Lewisham and Croydon; 17, Outreach and Support in South London (OASIS), Southwark and Lambeth; 18, Oxford Health NHS Foundation Trust Early Intervention Service; 19, Oldham early intervention team; 20, Rotherham Doncaster and South Humber (RDASH) Early Intervention; 21, Worthing Early Intervention in Psychosis Service; 22, Tower Hamlets Early Detection Service (THEDS); 23, Wakefield Early Intervention Team; 24, Widnes Early Intervention in Psychosis Service.
